# Junction formation of Cu_3_BiS_3_ investigated by Kelvin probe force microscopy and surface photovoltage measurements

**DOI:** 10.3762/bjnano.3.31

**Published:** 2012-03-23

**Authors:** Fredy Mesa, William Chamorro, William Vallejo, Robert Baier, Thomas Dittrich, Alexander Grimm, Martha C Lux-Steiner, Sascha Sadewasser

**Affiliations:** 1 Universidad Nacional de Colombia, Departamento de Física, Cra. 30 No 45-03, Bogotá, Colombia; 2 Universidad Libre, Departamento de Ciencias Básicas, Cra. 70 No 53-40, Bogotá, Colombia; 3 Universidad Nacional de Colombia, Departamento de Química, Cra. 30 No 45-03, Bogotá, Colombia; 4 Helmholtz-Zentrum für Materialien und Energie, Hahn-Meitner-Platz 1, 14109 Berlin, Germany; 5 International Iberian Nanotechnology Laboratory, Avda. Mestre José Veiga s/n, 4715-330 Braga, Portugal

**Keywords:** buffer layer, Cu_3_BiS_3_, Kelvin probe force microscopy, solar cells

## Abstract

Recently, the compound semiconductor Cu_3_BiS_3_ has been demonstrated to have a band gap of ~1.4 eV, well suited for photovoltaic energy harvesting. The preparation of polycrystalline thin films was successfully realized and now the junction formation to the n-type window needs to be developed. We present an investigation of the Cu_3_BiS_3_ absorber layer and the junction formation with CdS, ZnS and In_2_S_3_ buffer layers. Kelvin probe force microscopy shows the granular structure of the buffer layers with small grains of 20–100 nm, and a considerably smaller work-function distribution for In_2_S_3_ compared to that of CdS and ZnS. For In_2_S_3_ and CdS buffer layers the KPFM experiments indicate negatively charged Cu_3_BiS_3_ grain boundaries resulting from the deposition of the buffer layer. Macroscopic measurements of the surface photovoltage at variable excitation wavelength indicate the influence of defect states below the band gap on charge separation and a surface-defect passivation by the In_2_S_3_ buffer layer. Our findings indicate that Cu_3_BiS_3_ may become an interesting absorber material for thin-film solar cells; however, for photovoltaic application the band bending at the charge-selective contact has to be increased.

## Introduction

Thin-film solar cells based on absorbers made from Cu(In,Ga)Se_2_ [1] or CdTe [2] reach the highest efficiencies currently available. Both semiconductors are interesting for the application in solar cells because of their excellent absorption properties due to the direct band gap. With the current efforts towards a large-scale fabrication of such solar cells, problems may occur due to the limited availability of some of the constituents, such as In, Se, Cd or Te, and the respective toxicity of some of these elements. Therefore, current research efforts are exploring alternative, nonconventional, highly absorbing semiconductors to be used in thin-film solar cells. As one possible alternative, it was demonstrated recently that thin films of Cu_3_BiS_3_ can be prepared in a combination of chemical bath deposition and a sputtering process [3–4]. The band gap of these Cu_3_BiS_3_ thin films was shown to be ~1.4 eV [3], which makes them an excellent candidate for application in solar cells. It was also shown that thin films prepared by a coevaporation process present good structural and optical properties [5–6]. Recently, the potential of the Cu_3_BiS_3_/In_2_S_3_ heterojunction was investigated by surface photovoltage (SPV) and Hall-effect measurements, showing a passivation of surface defect states in the Cu_3_BiS_3_ by the In_2_S_3_ buffer layer and the formation of a photovoltaic active interface with a SPV of ~130 mV [7].

It is well known from the Cu(In,Ga)Se_2_ solar cells that a buffer layer is required between the n-ZnO window and the p-type absorber layer to reach high efficiency values [8]. Traditionally, CdS deposited by chemical bath deposition (CBD) has been used as a buffer layer to reach the highest efficiency figures. However, in recent years intensive research has been performed to avoid the toxic Cd-compound and implement a Cd-free buffer layer [9]. Successfully implemented materials include In_2_S_3_, ZnS, and Zn_1−_*_x_*Mg*_x_*O, deposited by a variety of techniques, such as chemical bath deposition, atomic layer deposition, ion layer gas reaction (ILGAR) deposition, evaporation, and spray deposition [9].

One interesting aspect of the above mentioned solar cell materials CdTe and Cu(In,Ga)Se_2_ is their high efficiency despite the abundance of grain boundaries (GBs). Scanning probe microscopy experiments have provided significant insight into the physics of grain boundaries on these materials [10]. Specifically, recent experiments provided evidence for the benign properties of the GBs [11–12], in agreement with previous theoretical work [13–14]. Also the influence of the buffer layer on the grain boundaries was addressed, providing evidence for a diffusion of sulfur from the CdS buffer layer into the grain boundaries of the Cu(In,Ga)Se_2_ absorber film [15–16].

In this work we present a comparative analysis of the nanoscale optoelectronic properties of Cu_3_BiS_3_ thin films and different buffer layers, investigated by KPFM, locally resolved SPV measurements, and macroscopic spectral SPV measurements.

## Results and Discussion

### Chemical surface analysis by X-ray photoelectron spectroscopy (XPS)

**Figure 1:**
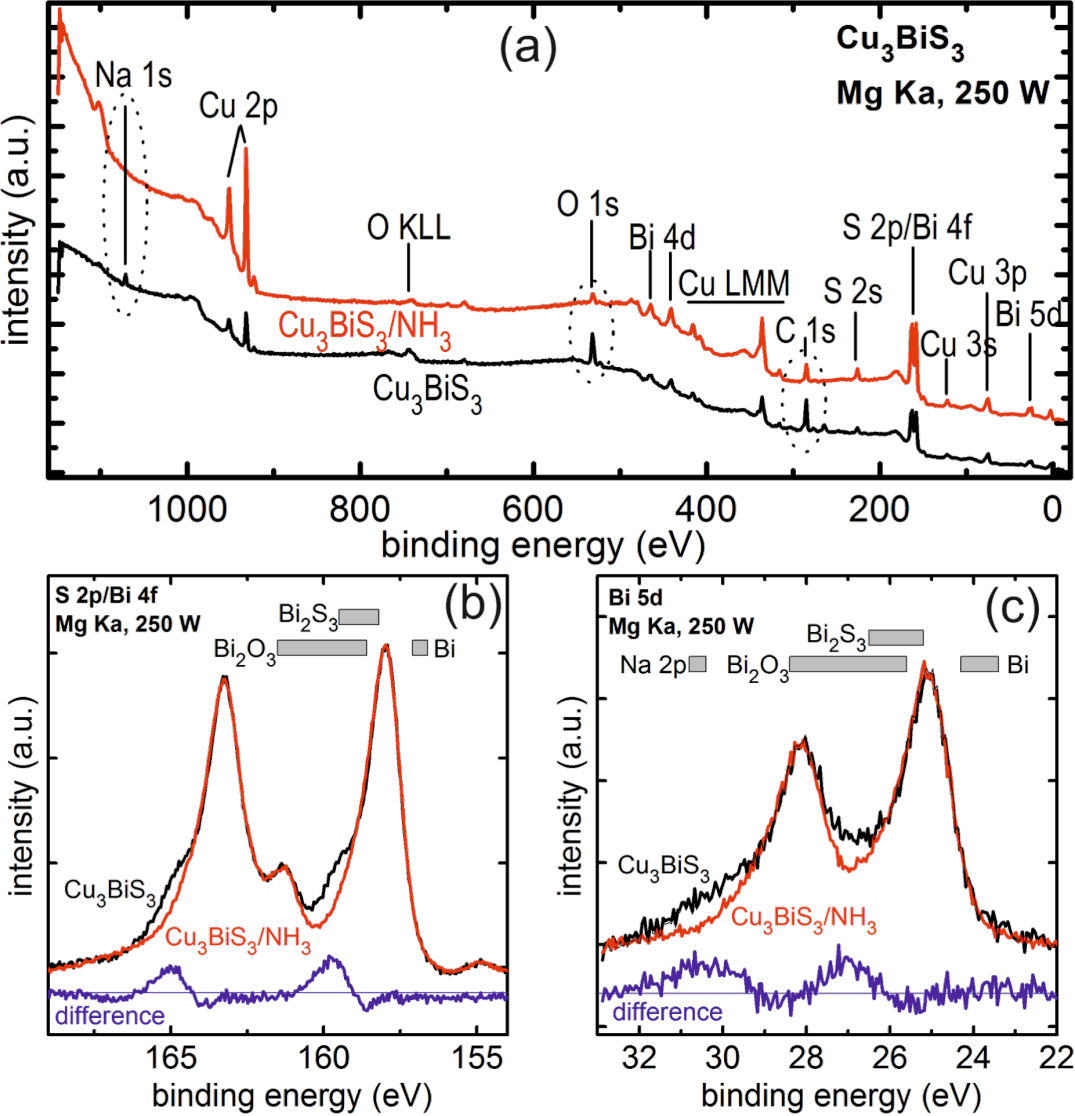
XPS measurements on Cu_3_BiS_3_ and Cu_3_BiS_3_ etched in NH_3_. (a) Overview spectrum showing that Na, oxides, and C contamination are effectively reduced by the NH_3_ etch. (b) Detailed spectrum of the S 2p and Bi 4f peaks showing the presence of Bi_2_O_3_ in the as-prepared Cu_3_BiS_3_ layer and its removal by the NH_3_ etch. (c) Detailed spectrum of the Bi 5d and Na 2p peak showing the presence of Bi_2_O_3_ and Na and their effective removal by the NH_3_ etch. The blue spectra represent the difference spectra between the as-prepared and the NH_3_-etched Cu_3_BiS_3_ samples.

For the validity and interpretation of surface-sensitive KPFM measurements, it is important to know the state of the surface of the examined sample. Surface oxidation can modify the work function of the sample and complicate data analysis. To clean the surface of Cu_3_BiS_3_ samples, we used an NH_3_ treatment prior to introduction into the ultrahigh-vacuum (UHV) system of the KPFM. To investigate the effect of this treatment, we analyzed samples exposed to the same NH_3_ treatment by XPS. Figure 1a shows an overview XPS spectrum of the as-prepared (lower curve) and NH_3_-etched (upper curve) Cu_3_BiS_3_ samples. The expected peaks of Cu, Bi and S are clearly visible. Additionally, the as-prepared Cu_3_BiS_3_ sample also shows signals of Na, C and O. Na presumably diffused out of the glass substrate, while C and O are a result of storage in air. The NH_3_ etch effectively removes the Na from the surface, while the peak heights of the C and O peaks are significantly reduced. More details about the chemical form in which oxygen is present can be inferred from the detail spectra shown in Figure 1b and Figure 1c. The as-prepared Cu_3_BiS_3_ sample shows clearly asymmetric peak shapes due to the presence of Bi_2_O_3_. It is also clear that the NH_3_ etch removes these oxide peaks to a large extent. This can be well seen by comparison of the difference spectra (blue line) with the expected range of the Bi_2_O_3_ peaks (gray boxes) [17]. The presence of an additional Bi_2_S_3_ phase cannot be completely excluded from the present measurements. However, the presence of elemental Bi can almost certainly be excluded. The analysis of the S 2s peak (not shown) additionally confirms the removal of sulfate phases by the NH_3_ treatment. Therefore, we can unambiguously confirm that the etched surface is in a state nearly free of oxides, which resembles the state of the Cu_3_BiS_3_ surface onto which the buffer layers from the chemical bath will be deposited.

### Surface characterization by Kelvin probe force microscopy (KPFM)

**Figure 2:**
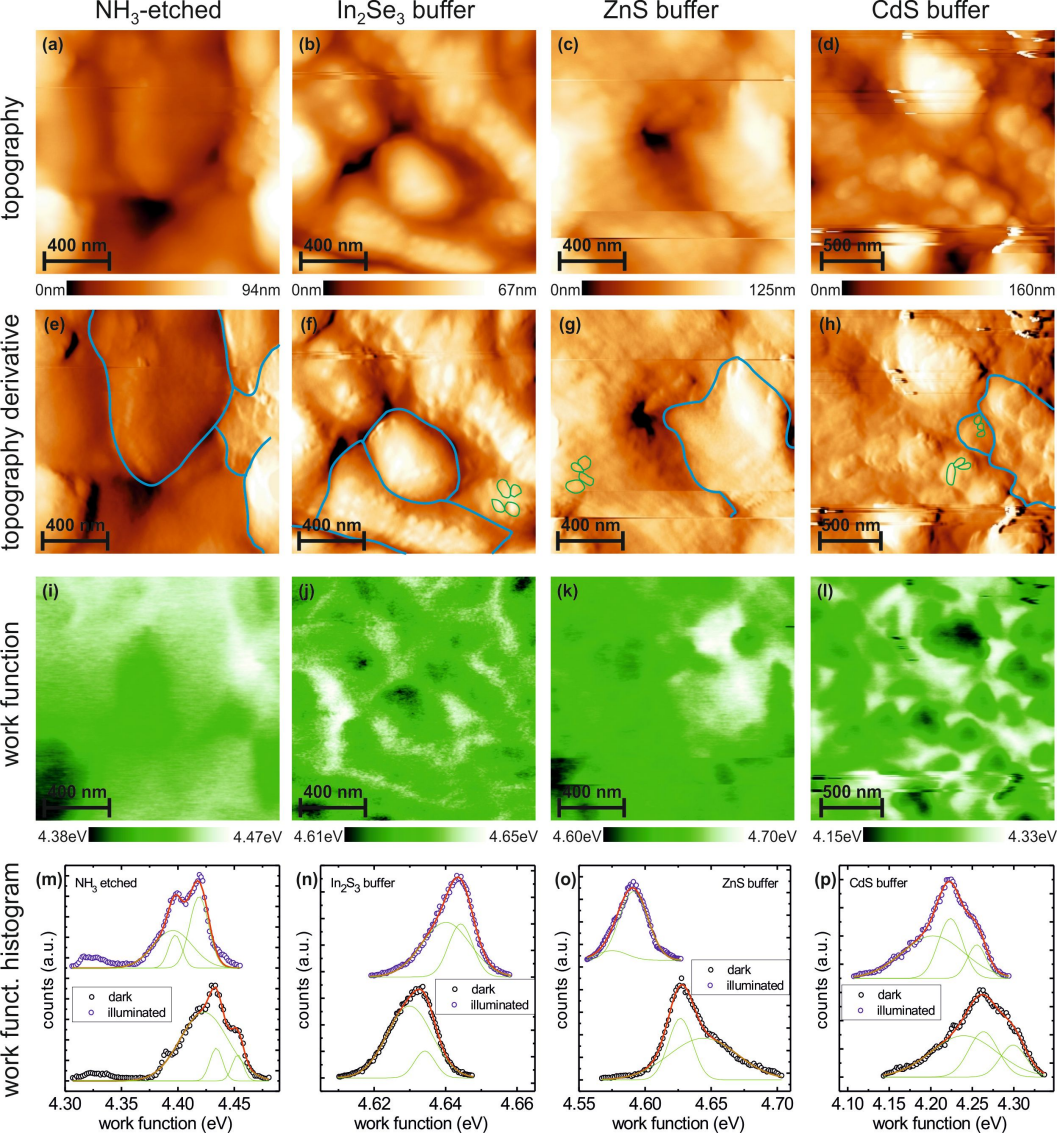
KPFM measurements of the (from left to right) NH_3_-etched Cu_3_BiS_3_, and Cu_3_BiS_3_ with the In_2_S_3_, ZnS and CdS buffer layers. The rows show, from top to bottom, the topography image, the derivative image of the topography, and the simultaneously recorded work-function image in the dark. The bottom row shows histograms of the work-function images in the dark (lower curve) and under illumination (upper curve) with respective Gaussian fits to describe the work function distributions.

In order to comparatively characterize the growth and electronic properties of the different buffers, we performed KPFM measurements on the Cu_3_BiS_3_ samples with all three buffer layers, and as a reference also on the pure Cu_3_BiS_3_ surface after NH_3_ etching. Figure 2 shows the results on all surfaces, in which the topography is shown in the upper row, the derivative of the topography in the second row, and the work-function image in the third row; a histogram displaying the work-function distribution in the dark and under illuminated conditions (red laser, λ = 675 nm, 70 mW/cm^2^) is shown in the bottom row. The columns show the surfaces of the NH_3_-treated Cu_3_BiS_3_, and the Cu_3_BiS_3_ with the In_2_S_3_, ZnS and CdS buffer layers, from left to right, respectively.

A wealth of information can be extracted from these KPFM images. The topography of all samples shows a granular structure with a grain size on the order of 300 to 600 nm, which corresponds to grains of the Cu_3_BiS_3_ film. The finer details of the topographic structure are more easily visible in the d*z*/d*x*-derivative images presented in the second row of Figure 2. Here a clear difference between the etched Cu_3_BiS_3_ sample and the samples with a deposited buffer layer is seen. The derivative image of the Cu_3_BiS_3_ sample shows smooth grain surfaces, whereas the corresponding images of the deposited buffer layers show small grains (exemplarily indicated by green lines) on top of the large grains of the Cu_3_BiS_3_ film (exemplarily indicated by blue lines). These smaller grains exhibit sizes on the order of 20 to 100 nm and can be attributed to the nanocrystalline nature of the deposited buffer layers.

The work-function images in the third row of Figure 2 provide additional information about the buffer layers and the Cu_3_BiS_3_ film. The presented images represent the raw data, shifted only by the constant work function of the tip. Due to the rough surface topography, sporadic tip changes could not be avoided (visible as the horizontal streaks). However, since the measured work function does not change abruptly at these tip changes, we can exclude a significant modification of the tip. The spread of the work-function distribution is therefore analyzed in the form of a work-function histogram, represented in the bottom row of Figure 2. The lower histogram represents the work-function measurement under dark conditions, while the upper histogram is measured for an illuminated sample.

**Figure 3:**
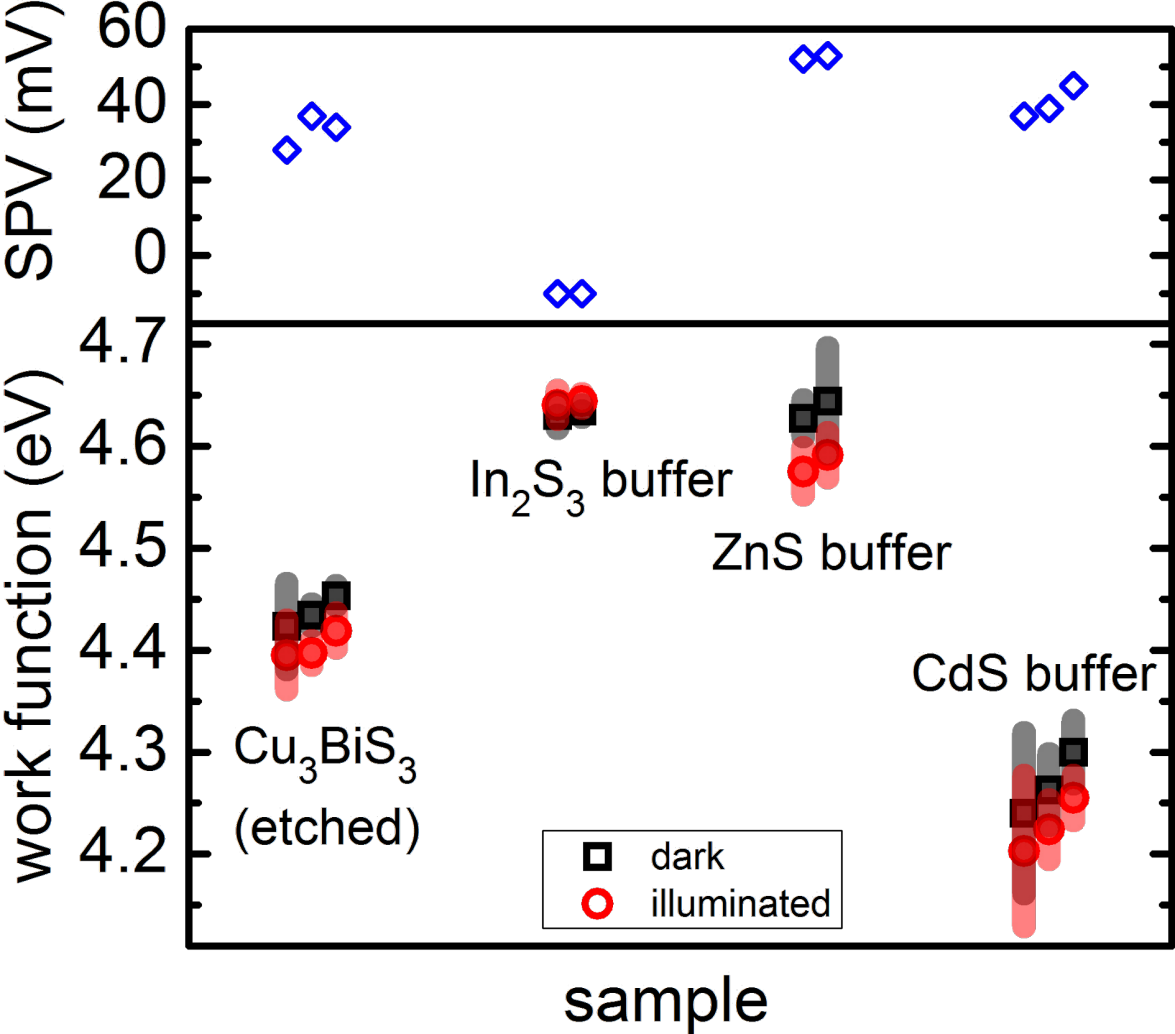
Overview of the measured work-function values and their distribution for all samples investigated. Black squares indicate values for measurements in the dark and red circles for measurements under illumination. The spread of the work function is indicated by the gray and light red bars around the data points.

The histograms can be fitted very well by two or three Gaussian distributions, where the center of each Gaussian distribution gives the most frequent value of the work function and the standard deviation σ gives a measure of the spread of the values. We would like to point out, that this spread is not to be confused with an error of measurement. It is a real distribution of work function values on the measured surface. The information thus extracted from the histograms is represented in a condensed form in Figure 3, showing for the different samples (on the *x*-axis) the center of the work function and the spread of the work function values. The values for each Gaussian curve are slightly offset for better visibility.

From Figure 3, it is clearly visible that the spreads of the work-function values for the etched Cu_3_BiS_3_ film and for the ZnS and CdS buffers on Cu_3_BiS_3_ are similar, in the range of 10 to 80 meV. In contrast to this, the spread of the Gaussian distribution for the sample with the In_2_S_3_ buffer is much smaller, only 6–13 meV. These results are in agreement with our previous measurements [7], from which we drew conclusions toward an effective passivation of the Cu_3_BiS_3_ surface by the In_2_S_3_ buffer layer.

Defining the surface photovoltage as the difference in work function between the dark and the illuminated state (SPV = Φ_dark_ − Φ_light_), we can compare the SPV between the different samples. This is shown in the upper panel of Figure 3. While the etched Cu_3_BiS_3_ surface and the CdS and ZnS buffer layers exhibit a positive SPV, only the In_2_S_3_ buffer layer exhibits a negative SPV. This corresponds to charge separation due to band bending at the internal Cu_3_BiS_3_/In_2_S_3_ interface when considering a p-type Cu_3_BiS_3_ and an n-type buffer layer. The positive SPV for the other buffer layers can be interpreted as a separation of holes towards the surface and consequently a reduction of upward band bending. More insight into the effects of the In_2_S_3_ buffer layer was obtained by a detailed investigation of this surface by macroscopic spectrally resolved SPV (see next section).

A special strength of KPFM is the possibility to obtain locally resolved work-function information, as displayed in Figure 2. Special attention can be devoted to the electronic structure of grain boundaries in these polycrystalline materials. For the NH_3_-etched Cu_3_BiS_3_ sample, only a weak correspondence can be observed between the topography (Figure 2a) and the work function (Figure 2i), indicating that the charge state at the grain boundaries is similar to that of the grain surface. On the In_2_S_3_ and CdS buffer layers, the situation is significantly different. The work function images in Figure 2j and Figure 2l show an increased work function at the position of the grain boundaries of the Cu_3_BiS_3_ film. From the comparison with the small granular structure observed from the images of the topography derivative, it is evident that the increased work function coincides with the grain boundaries of the underlying Cu_3_BiS_3_ film and not the In_2_S_3_ or CdS buffer layer. The more positive work function indicates negatively charged grain boundaries in the Cu_3_BiS_3_ film, in contrast to the NH_3_-etched film. The change in work function amounts to 10–20 meV for the In_2_S_3_ buffer layer and to 60–150 meV for the CdS buffer layer. The size of this upward band bending is in a similar range to the band bending observed for Cu(In,Ga)Se_2_ solar-cell absorbers [18]. In contrast to the In_2_S_3_ and CdS buffer layers, the ZnS buffer layer does not exhibit any significant contrast at the grain boundaries.

### Characterization using spectral surface photovoltage (SPV)

**Figure 4:**
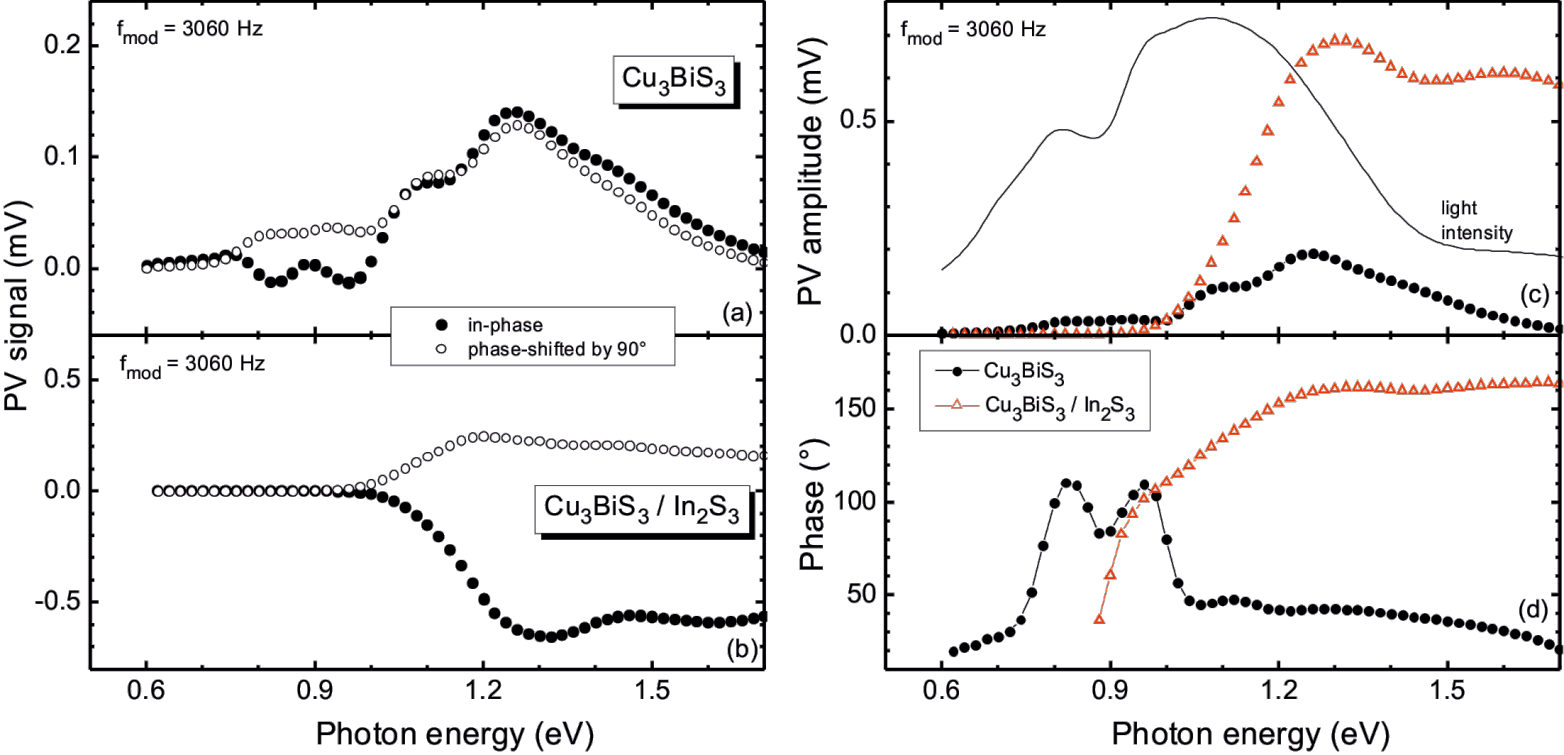
In-phase (solid circles) and 90°-phase-shifted (open circles) SPV spectra of (a) Cu_3_BiS_3_ and (b) Cu_3_BiS_3_/In_2_S_3_ samples at a modulation frequency of 3060 Hz. From these, spectra of the (c) PV amplitude and (d) phase angle for Cu_3_BiS_3_ (solid circles) and Cu_3_BiS_3_/In_2_S_3_ (open triangles) samples were derived. The solid line in (c) gives the light intensity.

Figure 4 shows the in-phase (also called *x*-signal) and 90°-phase-shifted (also called *y*-signal) photovoltage (PV) spectra of Cu_3_BiS_3_ (a) and Cu_3_BiS_3_/In_2_S_3_ (b) samples. The *x*-signal begins at photon energies significantly below the optical band gap of Cu_3_BiS_3_. For Cu_3_BiS_3_ the in-phase PV signal is initially positive and increases to about 13 µV with increasing photon energy. Before a strong increase of the signal up to 114 µV at photon energies around 1.24 eV, several sign changes are observed at about 0.79, 0.87, 0.91 and 0.99 eV and respective transitions in between. The *y*-signal shows similar characteristic transitions but without changes of sign.

For the Cu_3_BiS_3_/In_2_S_3_ sample the *x*- and *y*-signals exhibit negative and positive signs, respectively, and the signs do not change over the whole spectrum, in contrast to the Cu_3_BiS_3_ sample. The *x*- and *y*-signals begin at photon energies between 0.9 and 1.0 eV and reach maxima of −0.65 and 0.24 mV at 1.32 and 1.20 eV, respectively. The present case of a negative sign of the *x*-signal together with a positive sign of the *y*-signal can be interpreted as photogenerated electrons being preferentially separated towards the internal interface [19].

Several peaks or changes of the sign in the SPV spectra disappeared after the deposition of In_2_S_3_ on Cu_3_BiS_3_. This can be interpreted as the disappearance of surface defect states from which separation of photoexcited charge carriers is possible, i.e., chemical reactions at the Cu_3_BiS_3_/In_2_S_3_ interface lead to the passivation of surface-defect states in the band gap of Cu_3_BiS_3_.

Often it is useful to analyze the amplitude and phase angle instead of the *x*- and *y*-signals; the amplitude is defined as the square root of the sum of the squared *x*- and *y*-signals, and the cotangent of the phase angle is the ratio of the *x*- and *y*-signals. The amplitude and phase-angle spectra are shown in Figure 4c and Figure 4d for the Cu_3_BiS_3_ and Cu_3_BiS_3_/In_2_S_3_ samples. Additionally, the spectrum of the light intensity is shown for comparison. There are distinct shoulders and peaks in the amplitude spectra, which are related to the onset of electronic transitions from which charge separation is possible and that depend also on the light intensity. A normalization of SPV spectra to the light intensity or to the photon flux is usually impossible due to the fact that different processes, even with opposite direction of charge separation, may contribute to the SPV signal, making its response highly nonlinear. We would like to point out here that the PV amplitude in the present measurements is much smaller than that found in [7], since the present measurements were performed with modulated light and with a different light source/intensity. On the other hand, the behavior of the phase angle can help to distinguish dominant processes of charge separation and recombination. The peaks in the phase-angle spectrum of the Cu_3_BiS_3_ sample at 0.83, 0.96 and 1.12 eV can be interpreted, for example, as charge separation from deep defect states with the respective energies above the valence-band edge (excitation of electrons into unoccupied surface states) or below the conduction band edge (excitation of electrons from occupied surface states).

The phase angle of the Cu_3_BiS_3_/In_2_S_3_ sample changes strongly from about 35° at 0.88 eV to 161° at 1.32 eV and remains rather constant at higher photon energies, while a change from 160 to 165° can be distinguished between 1.46 and 1.7 eV. A spectral range with a nearly constant value of the phase angle can be understood as a spectral range for which the mechanisms of charge separation, transport and recombination remain similar. This can be the case, for example, at photon energies above the mobility gap if the diffusion length of photogenerated charge carriers is significantly shorter than the absorption length of the exciting light, and if the unmodulated quasi Fermi levels are not very different. Therefore, we can conclude that the mobility gap of Cu_3_BiS_3_ is about 1.3 eV. Furthermore, photogeneration of charge carriers from defect states leads to the appearance of mobile and immobile charge carriers and therefore to a change of response times. Usually the response times become longer and therefore a change of the phase angle from values close to 180° toward 90° can be expected; this was observed for the Cu_3_BiS_3_/In_2_S_3_ sample between 1.3 and 0.94 eV.

**Figure 5:**
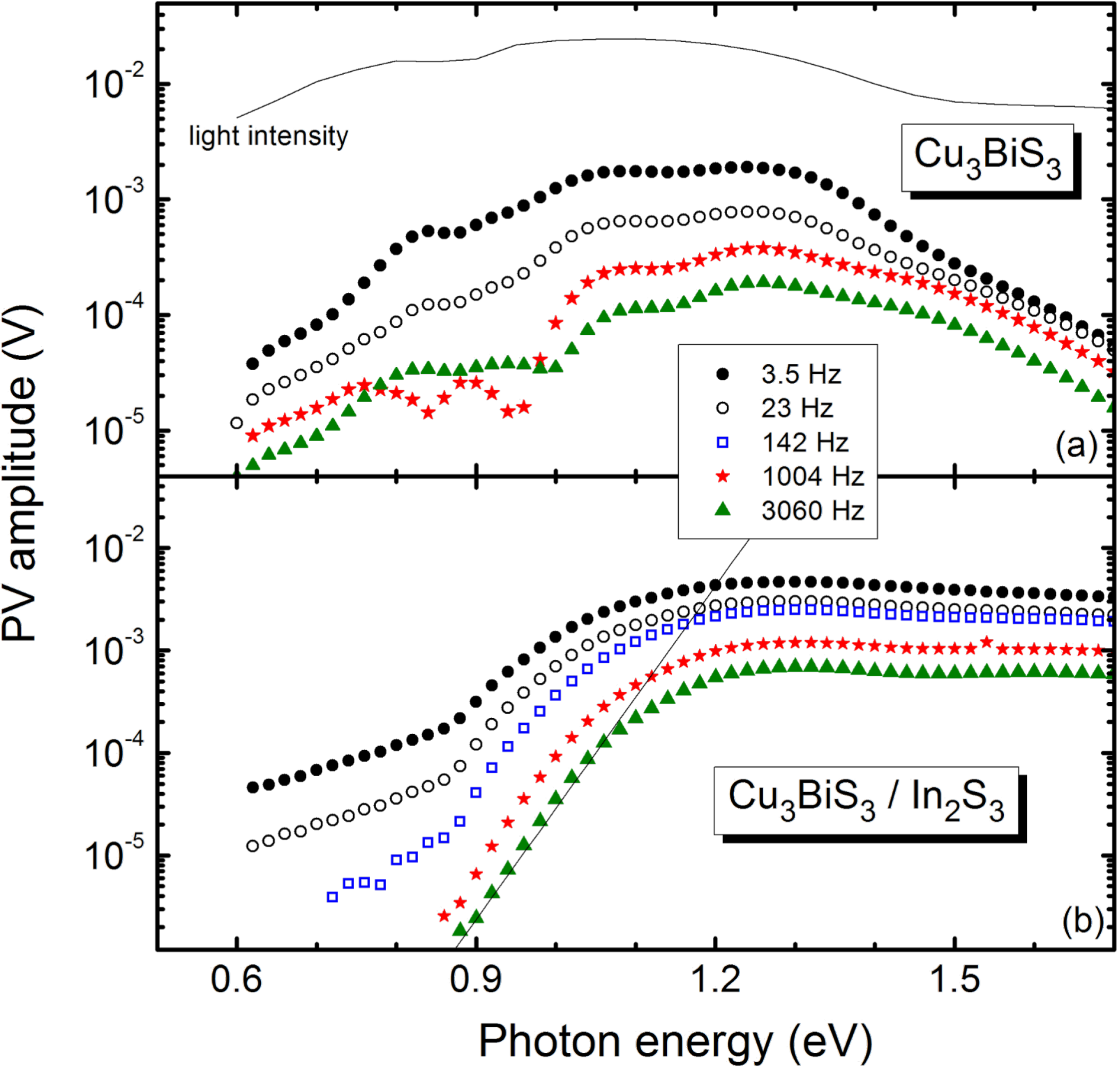
PV amplitude spectra of (a) Cu_3_BiS_3_ and (b) Cu_3_BiS_3_/In_2_S_3_ at modulation frequencies between 3.5 and 3060 Hz. The solid curve in the upper panel gives the light intensity. The straight line in the lower panel shows a fit to the data at 3060 Hz describing exponential tail states [20].

Defect states appear differently in PV spectra depending on the modulation frequency, due to the role of response times for different processes. PV amplitude spectra are depicted in Figure 5 for different modulation frequencies. Usually, PV amplitudes decrease with increasing modulation frequency. For the Cu_3_BiS_3_ sample signatures of the three relatively broad, deep defect levels were observed at all frequencies. We remark that dips in the amplitude spectra could appear, for example, when the sign of SPV signals changed. For better interpretation a correlation of SPV with photocurrent measurements at Cu_3_BiS_3_ layers would be helpful. Three characteristic spectral regions were distinguished for Cu_3_BiS_3_/In_2_S_3_ samples below 0.9 eV (deep defect states), between 0.9 and 1.1 eV (exponential tail states), and above 1.1–1.3 eV (photogeneration of mobile electrons and holes). The PV amplitude of the deep defect states vanishes at frequencies above 1 kHz for the Cu_3_BiS_3_/In_2_S_3_ samples. Disorder in the material leads to states in the band gap close to the band edges, resulting in an exponential decay of the SPV. The characteristic energy of these exponential tails can be estimated at about 40 meV for Cu_3_BiS_3_/In_2_S_3_ samples at a modulation frequency of 3060 Hz, based on the solid fit line [20].

## Conclusion

We have analyzed the formation of various buffer layers on Cu_3_BiS_3_ compound semiconductor films for applications as absorbers in solar cells. The In_2_S_3_, ZnS, and CdS buffer layers grow as small-grained films (grain size of 20–100 nm) on the Cu_3_BiS_3_ polycrystalline absorber. The deposition of In_2_S_3_ and CdS layers appears to charge the grain boundaries of the underlying Cu_3_BiS_3_ thin film negatively. In agreement with the narrow work-function distribution of the In_2_S_3_ buffer layer (width of 6–13 meV), the spectral surface photovoltage revealed a passivation of defect states at the absorber/buffer interface, evidenced by a reduction of the sub-band-gap SPV.

To develop the promising Cu_3_BiS_3_ semiconductor toward an efficient solar cell, future activities should include the investigation of the n-type window layer, i.e., ZnO. This additional n-type layer may also lead to an increased band bending at the pn-junction, thereby leading to better performing solar cells.

## Experimental

### Sample growth

Cu_3_BiS_3_ thin films were grown by coevaporation in two stages. In the first stage a Bi*_x_*S*_y_* layer is grown by simultaneous evaporation of Bi and S. In the second stage the Cu_3_BiS_3_ compound is formed by evaporation of Cu, in a sulfur environment, onto the Bi*_x_*S*_y_* layer, with the substrate temperature kept at 300 °C during the complete process [6]. Layers of Cu_3_BiS_3_ (thickness about 1 µm) were deposited on glass coated with Al, deposited by dc magnetron sputtering [21]. For analysis by Kelvin probe force microscopy (KPFM) [7], surface photovoltage (SPV), and X-ray photoemission spectroscopy (XPS), sample contact was established at the Al back contact.

CdS thin films were deposited onto the Cu_3_BiS_3_ layers from a solution containing thiourea (Scharlau) and cadmium chloride (CdCl_2_) (Merck) as sources of S^2−^ and Cd^2+^, respectively. The thickness of the films was ~80 nm, as measured with a Veeco Dektak 150 surface profiler. For specific experimental conditions see [22].

ZnS films were grown by coevaporation of metallic precursors evaporated from a tungsten boat for Zn and a tantalum effusion cell for sulfur. The substrate was heated to ~250 °C. A thickness monitor (Maxtec TM-400) with a quartz-crystal sensor was used to measure the deposition rate of Zn. The thickness of the films was ~120 nm, as measured with a Veeco Dektak 150 surface profiler. Details of the ZnS film preparation are given in [23].

In_2_S_3_ buffer layers were deposited by coevaporation of In and S on the substrate heated to ~300 °C. The deposition system consists of the same components mentioned above for the growth of films of ZnS. The thickness of the films was ~150 nm. Both, the ZnS and the In_2_S_3_ buffer layers were deposited on NH_3_-etched Cu_3_BiS_3_ films.

### X-ray photoelectron spectroscopy

The chemical surface condition of the as-prepared Cu_3_BiS_3_ and Cu_3_BiS_3_ etched with NH_3_ was analyzed in an ultrahigh-vacuum (UHV) chamber (“CISSY” at Helmholtz-Zentrum Berlin) by X-ray photoelectron spectroscopy [24]. Mg Kα (1253.6 eV) radiation from a SPECS XR 50 X-ray gun served as the excitation source. The emitted photoelectrons were detected by a CLAM 4 electron spectrometer from Thermo VG Scientific. For surface cleaning, the sample was etched in a 3% aqueous NH_3_ solution for 150 s at room temperature and transferred through air into the UHV system within less than 5 min.

### Kelvin probe force microscopy

Kelvin probe force microscopy measurements were performed in a modified Omicron UHV AFM/STM operating at room temperature and a base pressure <10^−10^ mbar [25], by using the amplitude-modulation technique (AM mode). We used PtIr-coated cantilevers (Nanosensors) with a first resonance frequency of ~75 kHz, measuring the contact potential difference (CPD) using the second resonance mode at ~450 kHz and an ac voltage of 100 mV. For detection of the cantilever oscillation a laser with λ = 980 nm was used, thus avoiding any excitation of the investigated samples. The work function of the surfaces was obtained from the measured CPD by calibration of the tip against a sample of highly oriented pyrolytic graphite with a known work function. The surface photovoltage was determined by illuminating the samples with red laser light (λ = 675 nm, 70 mW/cm^2^) and subtracting the work function in the dark [26]. Also for KPFM, samples were NH_3_ etched and transferred through air into the UHV system within less than 5 min.

### Spectral surface photovoltage

Spectral dependent SPV measurements were performed at −186 °C in the fixed-parallel-plate-capacitor arrangement [27]. A quartz prism monochromator with a halogen lamp was used for modulated excitation (mechanical chopper frequencies between 3.5 Hz and 3 kHz). SPV signals were measured by means of a high-impedance buffer with a dual-phase lock-in amplifier. Spectral SPV experiments were performed on untreated Cu_3_BiS_3_ samples.
